# Identification of Calpain Substrates by ORF Phage Display

**DOI:** 10.3390/molecules16021739

**Published:** 2011-02-21

**Authors:** Nora B. Caberoy, Gabriela Alvarado, Wei Li

**Affiliations:** Department of Ophthalmology, Bascom Palmer Eye Institute, Miller School of Medicine, University of Miami, Miami, FL 33136, USA

**Keywords:** calpain, protease, substrate, ORF phage display, calpastatin

## Abstract

Substrate identification is the key to defining molecular pathways or cellular processes regulated by proteases. Although phage display with random peptide libraries has been used to analyze substrate specificity of proteases, it is difficult to deduce endogenous substrates from mapped peptide motifs. Phage display with conventional cDNA libraries identifies high percentage of non-open reading frame (non-ORF) clones, which encode short unnatural peptides, owing to uncontrollable reading frames of cellular proteins. We recently developed ORF phage display to identify endogenous proteins with specific binding or functional activity with minimal reading frame problem. Here we used calpain 2 as a protease to demonstrate that ORF phage display is capable of identifying endogenous substrates and showed its advantage to re-verify and characterize the identified substrates without requiring pure substrate proteins. An ORF phage display cDNA library with C-terminal biotin was bound to immobilized streptavidin and released by cleavage with calpain 2. After three rounds of phage selection, eleven substrates were identified, including calpastatin of endogenous calpain inhibitor. These results suggest that ORF phage display is a valuable technology to identify endogenous substrates for proteases.

## 1. Introduction

Proteolysis is an important post-translational modification that regulates many fundamental biological processes, such as programmed cell death. Calpains are calcium-modulated cysteine proteases with at least 14 family members and are implicated in apoptosis of many tissues [1]. For example, Calpains are activated in ocular diseases, including retinal degeneration and neurodegeneration in glaucoma [2,3]. The two major isoforms, calpain 1 (µ-calpain) and calpain 2 (m-calpain), are ubiquitously expressed and activated by micromolar (3–50 µM) and millimolar (0.2–1.0 mM) calcium, respectively [1,2]. In general, these concentrations of Ca^2+^ are far greater than intracellular Ca^2+^ (<1 µM), suggesting that additional factors may be required [4]. Calpains 1 and 2 are heterodimers with a catalytic subunit and a regulatory subunit and have an endogenous inhibitor, calpastatin [4]. 

Substrate identification is the key to delineation of molecular pathways and cellular processes regulated by proteases. Phage display with random peptide libraries was previously used to define substrate specificity of proteases [5,6]. However, it is difficult to deduce endogenous substrates from mapped unnatural peptide motifs. Other groups used partially randomized sequences of known substrates to delineate substrate specificity of proteases by phage display [7,8,9]. Owing to uncontrollable protein reading frame, phage display with conventional cDNA libraries identified high percentage of non-open reading frame (non-ORF) clones [10,11], which encode short unnatural peptides with minimal implication to define endogenous substrates. We developed ORF phage display with minimal reading frame problem, proposed for the first time that ORF phage display could be used as a technology of functional proteomics to identify endogenous proteins with specific binding or functional activity [12], and demonstrated four versatile applications [13,14,15,16]. In this study we investigated the capacity of ORF phage display to identify unknown endogenous substrates for calpain 2 from an ORF cDNA library. Interestingly, calpastatin was identified as one of the substrates of calpain. The advantages and limitations of ORF phage display for identification of protease substrates are discussed and compared to other technologies. 

## 2. Results and Discussion

The ORF phage display system was developed from T7 phage vector with a biotinylation tag in the C-terminus of cDNA library proteins [13,17]. If cDNA library inserts are ORFs without in-frame stop codon, the tag is expressed and biotinylated by *E. coli* BirA ligase [18,19]. Thus, all ORF phage clones display biotin at the C-terminus of library proteins and are capable of binding to immobilized streptavidin. In contrast, non-ORF clones with stop codon(s) in cDNA library express no biotinylation tag and are eliminated through streptavidin binding. An ORF phage display cDNA library was constructed from mouse adult eye and >90% of clones in the streptavidin-enriched ORF library had ORF cDNA inserts [13]. The initial phage titer of this library was ~2 × 10^7^ pfu, which is expected to cover each ORF of ~23,000 genes in the human genome with an average of ~35 times per ORF in correct reading frames.

**Figure 1:**
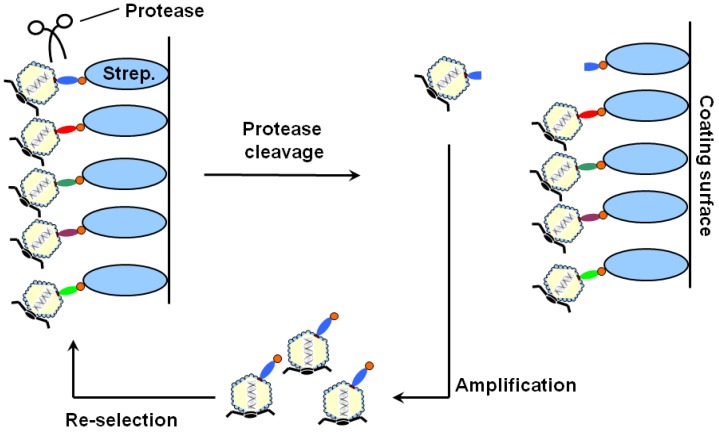
The scheme of ORF phage display for unbiased identification of protease substrates. ORF phage display cDNA library with C-terminal biotin bound to immobilized streptavidin on ELISA plates. After washing, bound phages were eluted by protease cleavage, amplified, and used as input for the next round of phage selection. Multi-round phage selection enriched substrate-encoding phage clones, which were individually analyzed for their release activity by protease cleavage.

The ORF cDNA library bound to immobilized streptavidin could be selectively cleaved at the displayed library substrates by protease of interest (Figure 1). Released clones could be amplified in host bacteria. The substrates and biotin will be restored and displayed on phage surface during the amplification. Multiple rounds of phage selection will selectively enriched phage clones displaying protease substrates according to their specificity for the protease.

**Figure 2:**
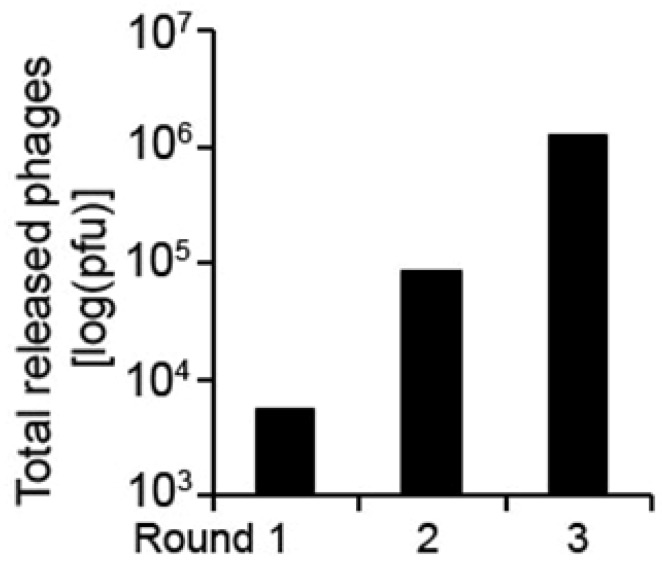
Enrichment of substrate-encoding phages. The ORF phage display cDNA library of mouse adult eye was enriched for 3 rounds as in Figure 1. The total eluted phages by calpain cleavage were quantified by plaque assay.

In this study, we used calpain 2 as a protease to test the above system to identify calpain substrates. Three rounds of phage selection resulted in more than ~300-fold increase in phage cleavability (Figure 2), suggesting that calpain-cleavable phages were substantially enriched. 

**Figure 3:**
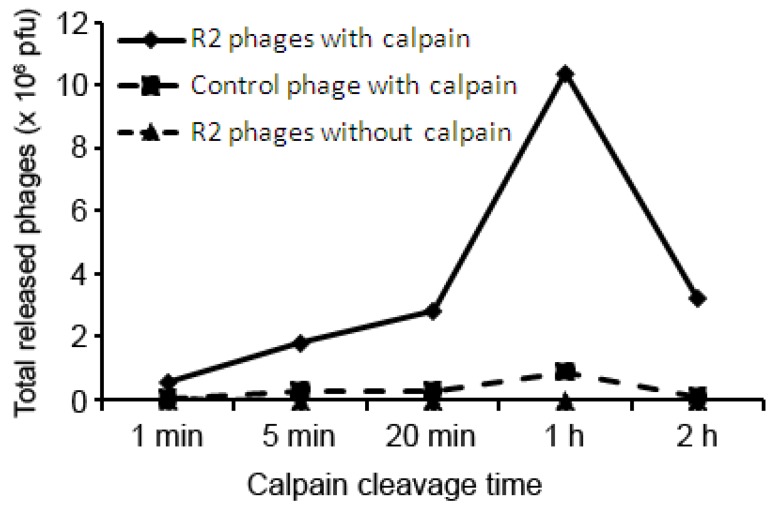
Time-dependent phage release by calpain cleavage. Enriched phages at round 2 and Biotin-phage bound to immobilized streptavidin, and eluted by calpain 2 or buffer control for indicated times. Eluted phages were quantified by plaque assay.

Enriched phage was specifically released by calpain cleavage in a time-dependent manner (Figure 3). However, lengthy digestion with calpain 2 resulted in decrease in released phages, possibly due to phage inactivation by calpain. Control Biotin-phage, which displays biotinylation tag with no other foreign cDNA insert [20], was included as a negative control to determine substrate-dependent phage release by calpain. The results indicated that the release of Biotin-phage was ~10-fold less active than the enriched library phages on average (Figure 3). 

**Figure 4:**
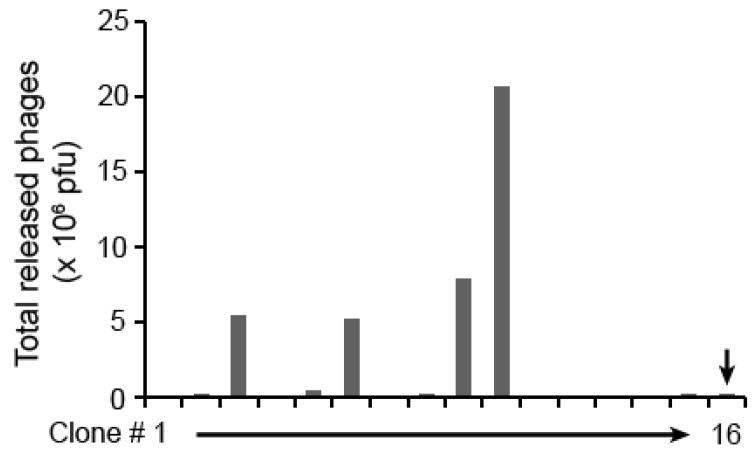
Screening for positive phage clones. Individual phage clones were randomly picked from the plates of enriched phages at round 2, and analyzed for their release activity by calpain cleavage. The eluted phages were quantified by plaque assay. A total of 43 phage clones were screened. Only 15 of them are showed here. The last clone (#16, indicated by arrow) is Biotin-phage as a negative control.

Excessive selection may lead to biased enrichment of clone species with relatively high substrate activity and marginalize those with moderate cleavable activity. This will result in decrease in phage clone diversity. We randomly picked individual phage clones from the plates of enriched phages at round 2 instead of round 3 for more clone diversity and analyzed their efficiency to be released by calpain cleavage. The results showed that 4 out of 15 randomly picked phage clones were highly cleavable by calpain (Figure 4). 

We screened a total of 43 phage clones and sequenced 13 with the highest cleavage activity by calpain. Eleven of them encoded real endogenous proteins in correct reading frames (Table 1). Two remaining clones were out-of-frame cDNA inserts. Interestingly, calpastatin was one of the identified substrates. Compared with biotin-phage, most of identified substrates showed ~16–90-fold increase in relative release activity by calpain. Calpastatin had ~436-fold increase in its relative release activity. We further analyze Ca^2+^-dependent release of identified phages by calpain. The result showed that calpain cleavage of all identified substrates increased by ~8–117-fold in the presence of 5 mM Ca^2+^ versus EDTA (Table 1). The release of calpastatin-phage by calpain showed ~54-fold increase in the presence of Ca^2+^.

Calpastatin is the only endogenous calpain inhibitor known so far, which reversibly inhibits calpain. The N-terminus of calpastatin is followed by four inhibitory domains (I, II, III and IV), each of which consists of three additional subdomains (A, B and C) [21]. The fragment encoded by the identified calpastatin-phage compassed subdomain C in domain III and subdomain A in domain IV (Table 1). Each inhibitory domain of calpastatin is capable of independently inhibiting one calpain molecule [21]. Previous studies showed that both the association of calpain with calpastatin and the dissociation of calpain from calpastatin seem to be dependent on Ca^2+^ [1,22]. It is unknown whether calcium-dependent cleavage of calpastatin by calpain will facilitate the dissociation of calpain-calpastatin for reactivation of the protease.

**Table 1:** Calpain-2 substrates.

Protein name	Accession number	Matched aa residues	Substrate relative cleavage activity	
			Calpain *vs.* control^a^	Ca2+ *vs.* EDTA^b^
Son cell proliferation protein (Son)	NM_178880	690L-833T	~90.6	~28.4
CCR4-NOT transcription complex, subunit 3 (Cnot3)	NM_146176	64T-232E	~16.7	~7.9
Skeletal muscle heavy polypeptides	Multiple^c^	Multiple	~35.1	~117.0
Ring finger protein 146 (Rnf146)	NM_026518	147V-338G	~62.4	~24.9
Catenin (cadherin associated protein), delta 2 (Ctnnd2)	NM_008729	1081S-11969H	~16.5	~16.8
Caldesmon 1 (Cald1)	NM_145575	121D-255R	~27.9	~14.1
Thymopoietin (Tmpo)	NM_001080132	89A-266E	~60.3	~43.7
Golgin subfamily a, 4 (Golga4)	NM_018748	1553K-1631K	~16.2	~9.1
Caldesmon 1 (Cald1)	NM_145575	131M-255R	~30.3	~11.1
Specc1	NM_001029936	163E-197Q	~36.4	~11.2
Calpastatin (Cast)	NM_009817	523P-623G	~436.1	~54.5

a. This is the ratio of phages released by calpain cleavage versus buffer control in the presence of 5 mM Ca^2+^; b. This is the ratio of phages released by calpain cleavage in the presence of 5 mM of Ca^2+^ or EDTA; c. This clone matches myosin heavy polypeptide 1, 2, 3, 4, 8 and 13 with various sequence homologies.

Several technologies can be used to map endogenous protease substrates, including mass spectrometry, phage display, yeast two-hybrid system, and other biochemical and cellular methods [23,24]. For example, mass spectrometry has been used to map calpain substrates in *Drosophila* [25] and calpain substrate specificity with a peptide library [26]. The major advantage of MS for substrate identification is that protease cleavage sites can be simultaneously defined [24]. In contrast, ORF phage display can only identify endogenous substrates, but not their cleavage sites. The major advantage of ORF phage display is that identified substrate-encoding phage clones can be used for re-verification and characterization without requiring the purification of substrate proteins. Purification of a large number of substrates identified by other technologies is time-consuming and labor-intensive. Our results showed that substrate specificity of identified phage clones can be conveniently quantified by phage plaque assay without special detection method. In addition, multiple rounds of phage selection and amplification will enrich less abundant clones for sensitive detection of substrates. However, thorough identification of all protease substrates will require time-consuming large-scale screening of individual phage clones. Although we developed a convenient colorimetric assay for high throughput screening and quantification of total bait-bound phages [13], this assay is not suitable for quantification of phages released by proteases. One of the options is to combine ORF phage display with next generation DNA sequencing [27,28,29] to globally map protease substrates. 

Like conventional phage display, ORF phage display has many versatile applications. We recently demonstrated its unique application for unbiased identification of eat-me signals for retinal pigment epithelium (RPE) phagocytosis, including tubby-like protein 1 (Tulp1) [15]. We independently validated Tulp1 for its functional activity to facilitate RPE phagocytosis and further characterized MerTK as its phagocytic receptor with receptor activation and intracellular signaling cascades [30]. These results suggest that ORF phage display is a valid technology of functional proteomics and is capable of identifying biologically relevant proteins with specific binding or functional activities. In addition, we showed that ORF phage display is capable of identifying endogenous binding proteins with an accuracy rate of ~71% [13], comparable to yeast two-hybrid system and mass spectrometry. Binding proteins for non-protein bait molecule, such as phosphatidylserine, were identified by ORF phage display and independently verified [14]. The T7 phage-based ORF phage display without requiring the displayed proteins to be secreted through *E. coli* membrane [13,17], as required in filamentous phage [31], is particularly suitable for unbiased display of mammalian proteins [32,33]. Together with our other studies, the results of this study suggest that ORF phage display is a versatile technology of functional proteomics and is applicable to unbiased identification of protease substrates. 

## 3. Experimental

### 3.1. General

Rat recombinant calpain 2 was purchased from Novagen (Madison, WI, USA). ORF phage display cDNA library was described previously [13]. All other reagents were purchased from Sigma (St. Louis, MO, USA). 

### 3.2. Phage selection

ELISA plates (Corning Life Science; #2592) were coated with streptavidin (10 µg/mL in PBS) at 4 °C overnight, blocked for 1 h with 1% polyvinyl alcohol (PVA) and incubated with the phage library (~1 × 10^10^ pfu/mL, 0.1 mL/well) in the presence of 0.1% Tween-20 for 1 h at room temperature [13]. The wells were washed 6 min for 6 times with PBST, followed by calpain buffer (50 mM Tris-HCl, pH 7.6, 150 mM NaCl, 5 mM β-mercaptoethanol, 5 mM CaCl_2_) for 6 min twice. Bound phages in the well were incubated with calpain 2 (~1 unit/100 µL/well) in the calpain buffer for 1 h at room temperature. The eluted phages were transferred to a new tube and mixed with final concentration of 10 mM EDTA to quench calpain activity. An aliquot of eluted phages was quantified by plaque assay to determine total eluted phages. The remaining phages were amplified in BLT5615 bacteria. The lysate was used as input for the next round of selection. In the subsequent selection, phages were eluted by proteases for 15 min to improve substrate specificity. A total of 3 rounds of selection were performed. 

### 3.3. Clone screening

Individual phage clones were randomly picked from the plates of enriched phages at round 2, amplified, and bound to immobilized streptavidin on ELISA plates. After washing, bound phages were incubated with calpain 2 for 15 min as above. Released phages were quantified by plaque assay [34]. Biotin-phage [20] was included as a negative control. Positive clones with at least 10-fold increase in release activity by calpain was re-verified and identified by DNA sequencing, as described [13]. 

### 3.4. Cleavage efficiency

All identified phage clones were analyzed for their cleavage activity by calpain 2 in the presence of Ca^2+^ or EDTA (5 mM) for 15 min. Biotin-phage was included as a negative control. Released phages were quantified by plaque assay. The substrate relative release activity was defined as total phages released by calpain cleavage versus buffer control in the presence of 5 mM Ca^2+^. The calcium dependence was defined as total phages released by calpain cleavage in the presence of 5 mM of Ca^2+^ versus EDTA. 

## 4. Conclusions

The results of this study suggest that ORF phage display is capable of identifying real endogenous substrates for proteases with minimal reading frame problem. The advantages of ORF phage are the convenient re-verification and characterization of identified substrates without requiring for pure proteins.
